# Influence of the Undercut Anchor Head Angle on the Propagation of the Failure Zone of the Rock Medium—Part II

**DOI:** 10.3390/ma14143880

**Published:** 2021-07-12

**Authors:** Józef Jonak, Robert Karpiński, Andrzej Wójcik

**Affiliations:** Department of Machine Design and Mechatronics, Faculty of Mechanical Engineering, Lublin University of Technology, Nadbystrzycka 36, 20-618 Lublin, Poland; a.wojcik@pollub.pl

**Keywords:** undercut anchor, anchor head angle, FEM analysis, rock breakout failure, rock cone failure, concrete cone failure wear, mining extracting tool

## Abstract

Problems concerning the influence of the geometric parameters of an undercutting anchor on the range of the failure zone of rock medium during the pulling out of the anchor constitute one of the aspects that arouse the interest of authors due to attempts to use undercutting anchors in the process of rock lump separation. This method is considered an alternative to the existing methods of separation, especially in special cases of mining technologies. This article presents the results of numerical investigations into the effect of changes in the head geometry that occur as a result of wear on the conical part of the undercutting anchor and the extent of failure of the rock medium during its pulling out. This is an extension of considerations presented in previous work, where special attention was paid to the influence of potential errors in anchor installation, leading to changes in head geometry and, consequently, to changes in the extent of the failure zone of the rock medium. As a result, significant changes in the volume of the detached rock masses are observed. This study shows that the increasing surface friction of the stripping anchor head leads to a decrease in the angle of the undercutting head. As a result, the extent of the failure zone measured on the free rock surface increases, the value of the angle of the failure cone at the initial stage of the stripping decreases, and the deformation of rock in the plane perpendicular to the anchor axis increases.

## 1. Introduction

The use of numerical modeling in conjunction with the results obtained in experimental studies enables a detailed understanding of the actual behavior of engineering structures and their optimization [1,2,3,4], as well as the reduction of costs associated with conducting experiments. Fracture problems of rock media and concrete (especially steel-reinforced ones) are currently analyzed using Finite Element Method (FEM) algorithms [5], deterministic and probabilistic methods [6,7] and ABAQUS programs (e.g., [8]).

The authors used the Extended Finite Element Method (X-FEM) of crack propagation. The Extended Finite Element Method is a finite element method of crack simulation that is independent of the element mesh. Modifying the shape function of the element allows the finite element to be separated at any location, so that the element mesh does not have to be too dense. Another commonly used method to simulate cracking in brittle material is the continuous-discontinuous method, which involves the introduction of a weakening material in the crack propagation region. This method is particularly popular for modeling concrete cracking. This smeared crack method is used in procedures implemented in commercial FEA codes (ANSYS and Simulia Abaqus). There exist a variety of FEM-based strategies to simulate cracks other than the XFEM, for instance, the phase-field method: residual stress as a fracture toughening mechanism [9], or, for example, the node release technique [10].

**Figure 1:**
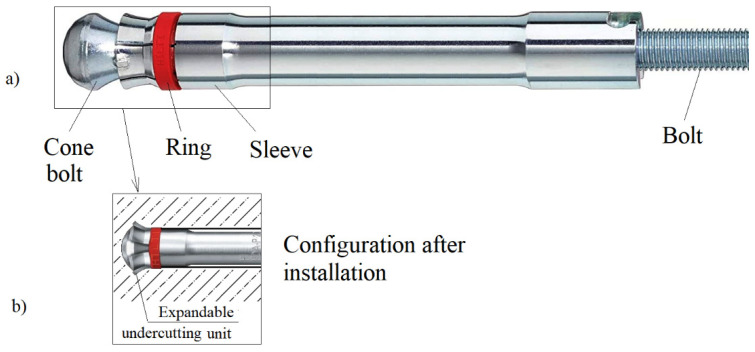
Undercut anchors type Hilti HAD, configuration (**a**) before installation (**b**) after installation.

In typical applications, the undercut anchor (Figure 1) is used in the technology of embedding steel construction elements, in concrete engineering structures [11,12,13]. This type of anchor, in installation form (Figure 1a), is embedded in a hole previously drilled in the concrete. The concrete is then undercut by the simultaneous rotation of the sleeve and its successive screwing onto the threaded part. The expanding sleeve elements supported on the “conical” anchor head then make an undercut in the concrete (Figure 1b), leading to the installation of the anchor. These elements are additionally reinforced with tungsten carbide plates to facilitate undercutting in hard concrete and to increase its durability.

**Figure 2:**
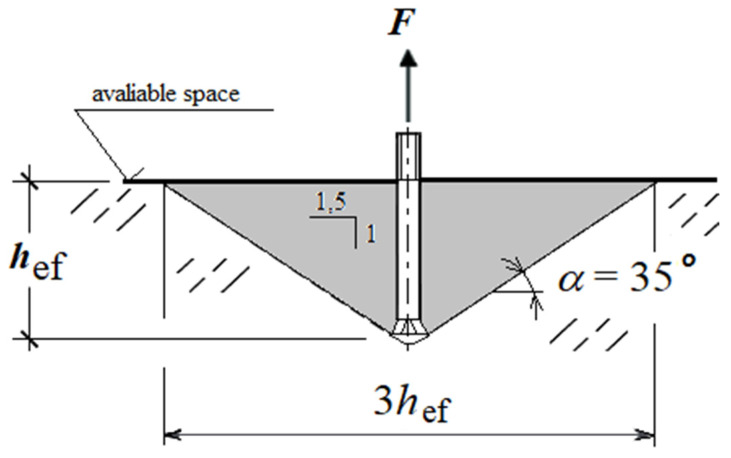
Model of the concrete cone failure under the action of the undercut anchor, according to the CCD method: *h*_ef_—effective anchoring depth; α—the angle of failure cone (pseudo-cone of failure).

At present, a simplified model of concrete failure in the form of the so-called cone of failure (Figure 2), developed by a number of scientists and practitioners, e.g., [14,15], is used. The material is treated here as a homogeneous medium. The characteristic feature of the CCD (Concrete Capacity Design) method [16] is the simplified form of the medium failure, where the range of failure (radius of cone base) measured at the free surface is 1.5 times greater than the effective anchoring depth *h*_ef_ (Figure 2).

Detailed recommendations on anchor setting to ensure the required load capacity of an anchor or anchor assembly are given in, e.g., [14]. A number of theoretical studies (including FE analysis) have been conducted on the mechanics of concrete failure under the action of anchors of various designs [17,18]. In earlier years, issues were analyzed mainly based on linear elastic fracture mechanics. More recently, concrete has been treated as a mainly non-linear material, i.e., an elastic-plastic medium due to the formation of micro-damage and the change in stiffness as the cohesion of the medium is destroyed. Both experimental studies and theoretical considerations (3D models) also draw attention to the contribution of tearing (circumferential) stresses in the plane perpendicular to the anchor head axis. In the case of undercutting heads, these stresses are related to the action of the tapered undercutting head, and it is suggested that they lead to a reduction in the anchor pull-out force. In 2D models, this aspect is difficult to identify and describe. Cross-sectional coverage of the issues of both 2D and 3D modelling as well as possibilities and limitations resulting from the applied approach to the subject of anchor interaction with the medium (concrete) can be found in publications such as [19,20,21]. For a given configuration of anchor pull-out device support arrangement and effective anchorage depth *h*_ef_, e.g., instability of crack propagation has been found, especially in the cover phase (i.e., from the moment when the stress in the compression or tension zone reaches a critical value), e.g., [19,21]. Even today, with the use of FEM (Finite Element Method) systems, one of the most important issues to be solved is to develop a model of the material of the medium (in which the anchors are embedded) which would satisfactorily describe the course of the failure process of its structure. For concretes, in ABAQUS, here are three constitutive models for defining the inelastic behavior of concrete: the concrete smeared cracking model (CSCM), concrete damaged plasticity (CDP) model, and the brittle cracking concrete (BCC) model. Plasticity conditions of the Mohr–Coulomb or Drucker–Prager failure criterion type or their modifications are used to describe rock properties (e.g., [22]). In engineering practice, there is a significant heterogeneity in the structure of natural materials in relation to concretes. As in the case of concrete, it is also difficult to determine unambiguously the value of the friction coefficient in the contact zone between the undercutting head and the rock, because in many cases, before the actual detachment occurs, the rock is crushed, causing a significant change in the friction and contact conditions. The authors of a number of studies on anchoring give a fairly wide range of values for the coefficient of friction of the medium against the steel or carbide from which the mining tool is made [23,24,25].

Studies [26,27,28] have extended the use of undercut anchors to the area of targeted, controllable failure of rock structures under non-typical conditions, e.g., during rock clearing in areas where mechanical mining with typical technologies is not possible. Multiple uses of the undercut anchors are also envisaged in different rock formations that may significantly differ in compressive and fracture toughness as well as the coefficient of friction of the rock against the undercut anchors. During the repeated installation of anchors, these parameters can have a significant influence on the intensity of wear of the conical head of the anchor and change the working parameters of this element. The essence of research [26], for a given seating depth (*h*_ef_) (Figure 2), was, among other things, to determine the value of anchor pull-out forces depending on the mechanical parameters of rock formations and to find out the mechanism of failure of the medium structure during the pull-out of a single anchor as well as a set of anchors. In studies [13,29,30], the course of the propagation trajectory of the surface (fracture) of the failure was analyzed, as it has a decisive influence on the volume of a potential rock mass to be peeled off. It was assumed that the latter aspect, apart from the value of the anchor pull-out force, can be used to assess the effectiveness of the proposed technology of rock mass detachment and the failure of the rock mass structure, in the process of the controlled and targeted pull-out of undercutting anchors.

In typical applications, seating of the anchor in concrete is followed by installation of the necessary infrastructure. In such cases, the anchor is embedded once for its entire lifetime and the process of potential wear of the undercutting surface is not taken into account here.

In Part I of this paper, the influence of the inaccuracy of anchor installation in the borehole (inaccurate undercutting of rock) on the formation of the extent of destruction of the rock medium was analyzed. Potential installation inaccuracy leads to oscillation of the undercutting head angle at the final stage of its propagation in the hole. Consequently, this translates into changes in the extent of the rock destruction zone. Ultimately, this can lead to disturbances in the interaction of the rock-destroying cones and reduced efficiency of the mining process. Part II analyzed the effect of anchor head wear on the extent of the failure zone. The study showed that with proper anchor installation, the increase in anchor wear mainly translates into a decrease in anchor head angle. This leads to an increase in the extent of the rock failure zone (along the free surface of the rock). Numerous experimental studies have shown that this process is accompanied by the formation of radial cracks (in the plane perpendicular to the anchor axis). This is accompanied by a decrease in the pull-out force of the anchor. However, this aspect requires further research to optimize the rock crushing process in terms of the energy consumed in the process and the forces required to pull out the anchor.

**Figure 3:**
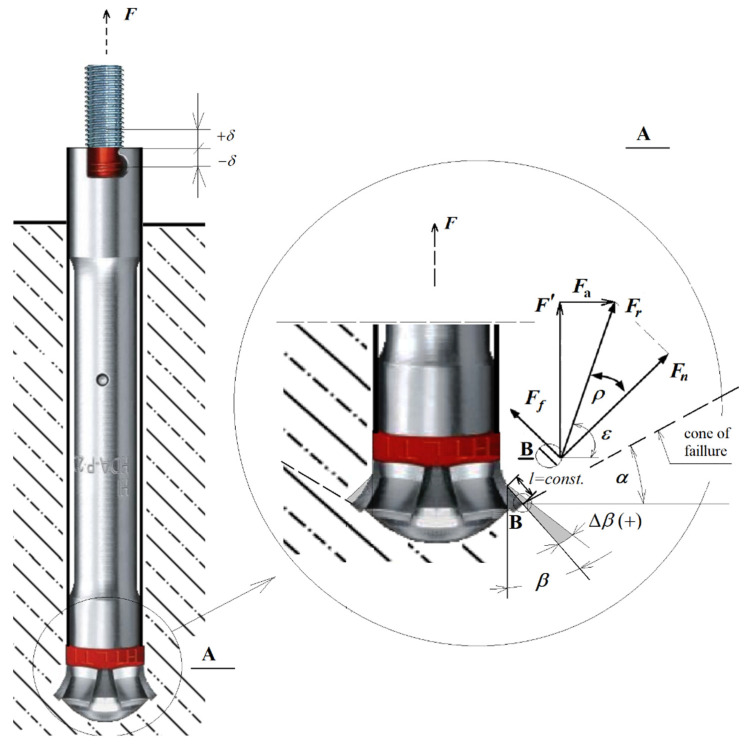
Force distribution on the undercutting element of the anchor: *F*—anchor pull-out force, *F*’—a component of the anchor pull-out force per undercutting element, *F*_f_—friction force of anchor element against the rock, *F*_n_—normal component to the surface of the undercutting element, *F*_a_—circumferential component, *F*_r_—resultant force of the action of the undercutting element on the rock, *ρ*—the angle of friction of the rock against the undercutting element, *ε*—the angle of the resultant force *F*_r_, α—the angle of the pseudo-cone of failure, *β*—the angle of rock cut with the undercutting head element equal to the angle of the cone of the undercutting head (more precisely, the angle of half the head cone), *ε*—angle of the resultant force on a single undercutting element (*ε* = *β + ρ*), *δ* (±)—deviation of the marker’s position from the recommended nominal position affecting the change of the undercutting head angle Δ*β*, and *l* = const.—length of the head cone.

The undercut angle *β* (Figure 3) is determined during the embedding of the anchor by observing the required installation dimensions imposed by the manufacturer, e.g., Hilti [12].

In the first part of the study [31], the inaccuracy of the anchor installation in the hole during embedding, on the formation of the angle of the undercutting head *β* was given special attention. This leads to changes in the angle of the undercutting head *β*, resulting in changes in the extent of the failure zone of the rock medium, including changes in the angle of the failure cone α. Keeping in mind the details of the Hilti-type anchor design, it was assumed that the length of the cone sidewall forming *l*= constants (Figure 3). It was assumed that inaccuracy of anchor installation (deviation of marker position from the nominal position resulting from anchor design—parameter *δ*, Figure 3) translates into a potential change of undercutting head angle Δ*β*. As a result of the analysis, it was found [30] that this has a significant impact on the crack trajectory and the extent of the failure trefoil. Increasing the angle of undercut *β* (the same as the angle of undercutting head) has an influence on limiting the range of detachment measured on the free rock surface. 

During research [26], it was found that after installation the angle *β* value for new M12 anchors is about 35°, while for larger, M20 anchors it is about 30°. During repeated installation and pull-out of the same anchor, significant changes were found in the geometry of the undercutting head due to surface wear. The intensity of this process was dependent on the mechanical parameters of the rock medium. The dulling and wear of mining tools operating in contact with rocky media is a common phenomenon, e.g., [32,33], and leads to changes in the geometry of the tool cutting edge [31], an increase in its load and vibrations of the mining heads [34,35,36,37]. Tribological interactions between rock and tool are analyzed using FEM and DEM (Discrete Element Method) [38,39,40].

**Figure 4:**
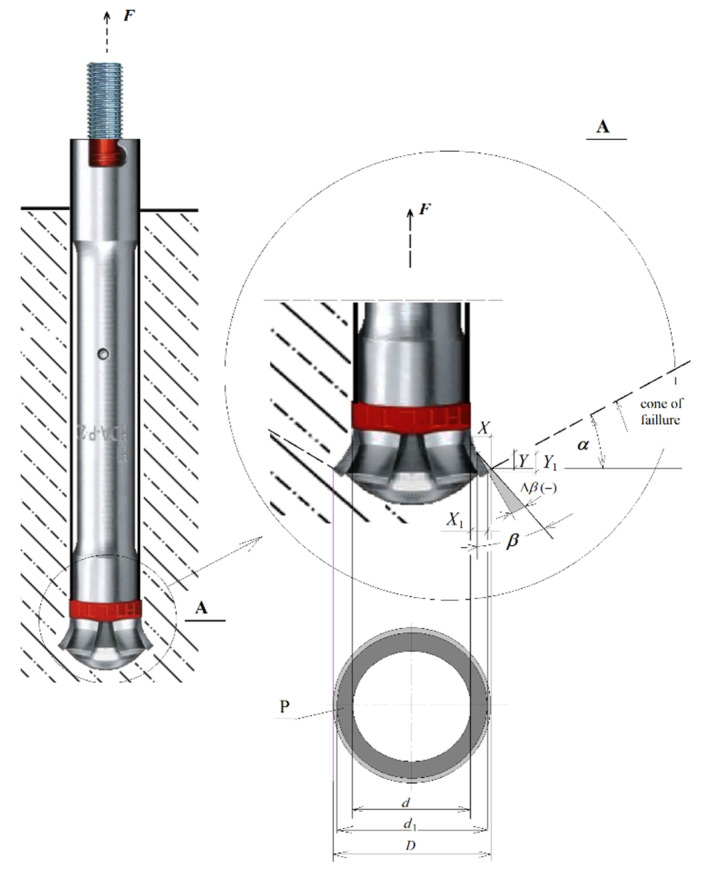
*F*—anchor pull-out force, *β*—the angle of rock cut with the undercutting head element equal to the angle of the cone of the undercutting head (more precisely, the angle of half the head cone), *d*—diameter of the anchor mounting hole, *D*—diameter of the undercutting of the rock with the head during assembly according to the producer’s recommendations, *d*_1_—undercutting diameter in case of wear undercutting head, Δ*β*—the change of the undercutting head angle, P—active surface of the head in contact with rock (projection of the conical surface of the head in contact with rock on a surface perpendicular to the anchor axis), and *x*, *x*_1_, *y*, *y*_1_—coordinates of the base corner of the conical part of the head.

The operation of the undercut anchor head under heavy load and in the presence of friction leads to intensive wear and tear and, as a result, to a decrease in the angle of the rock cut (Δ*β*, Figure 4). The characteristic dimensions of the rock undercut are also changed (values of *x* and *x*_1_ parameters in Figure 4). The size of the surface, which is important for the transferring of pressure in contact between rock and anchor, also changes on the conical surface of the anchor head. The projection of the conical head surface in contact with the rock to a surface perpendicular to the anchor axis (P, Figure 4) changes.

In the interaction of anchors with concrete, special attention is paid to the actual size of the active surface of the anchor (P—Figure 4) (e.g., [41,42,43]). Its size affects the load capacity of the anchor and the distribution of pressure in the contact zone with the rock, leading to the potential crushing of the medium in the contact zone. These processes are also reflected in the presented analyses.

A change in the head geometry Δ*β* (due to increasing wear) as well as a change in the friction coefficient specific for a given rock formation may lead to a change in the required anchor pull-out force resulting from existing calculation models as well as a change in the extent of the material failure zone. The latter aspect has a bearing on the volume of the material to be stripped and, consequently, on the efficiency of the proposed new stripping technology. Therefore, it is one of the criteria for the assessment of the efficiency of separation in the proposed new separation technology. Hence, there is also an interest in shaping the range of the medium failure zone under the action of the considered anchor construction, depending on the level of wear of the head and the presented analysis of the problem. The purpose of this study is to investigate the effect of changes in geometric parameters of the anchor head due to tool wear on the extent and course of rock failure.

## 2. Materials and Methods

In order to trace the effect of decreasing angle *β* (due to the progressive wear of the anchor head), the fracture trajectory at the initial stage of failure propagation and several variants of anchor head interaction with different head angles *β* (identical to the rock undercut angle) were analyzed. The analysis was carried out using the FEM system ABAQUS and the results are presented below. An important parameter here is the angle α (Figure 4), at which the damage starts to develop in the initial stage of failure (this stage of failure was taken into account because it is accompanied by the stable development of the crack/failure surface, i.e., in the range of linear dependence *σ*-*ε* for the considered rocks).

**Figure 5:**
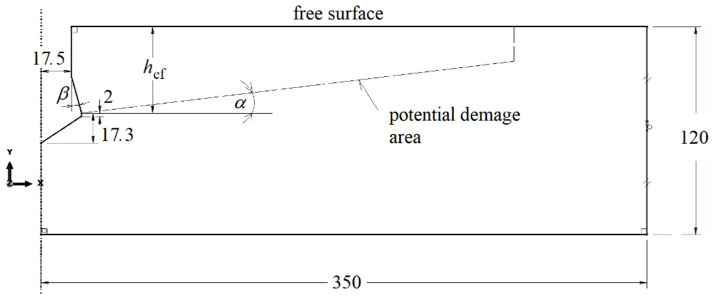
Axisymmetric model of anchor head interaction with rock, M20 anchor, dimensions in [mm].

An axisymmetric model of the interaction of the anchor head with the rock was used (Figure 5). An effective setting depth was assumed to be *h*_ef_ = 50 mm. Dimensions of the model: width (radius of the axisymmetric model)—350 mm, height—120 mm. The distance of the anchor axis from the vertical side restraint of the model was, therefore, 7 times greater than the effective anchorage depth. The potential impact of the supports observed in the “pull-out” method was reduced to a minimum (“free break-out test”). The height of the model (120 mm) was 2.4 greater than the anchoring depth. The influence of the ratio of the thickness of the rock medium layer to the anchoring depth and the influence of the anchoring depth on the extent of the failure zone were not analyzed. The wear rate of the anchor head was simulated by changing the angle of the anchor head *β*. In the simulation, the values of this angle were assumed to be equal to, respectively: *β* = 30°, 25°, 20° and 15°. The structure of the undercutting head was modelled in a certain simplification. The passage of the conical part (with its form in the coordinate system at the angle *β* to the anchor axis) to a cylindrical part with a height of 2 mm (as in Figure 5) was assumed. The further part of the outline of the model in the part under the anchor is a reflection of the bottom of the hole made with a dedicated drill in the procedure of preparing the anchor for seating in the hole.

It was also assumed that the angle value *β* = 30° corresponds to the taper angle of the new head. Progressive dulling/wear of the anchor head leads to a decrease in this angle during operation, which is reflected in the conducted FEM analysis.

The numerical analysis was carried out using the eXtended Finite Element Method (XFEM) in ABAQUS (Abaqus 2019, Dassault Systemes Simulia Corporation, Velizy Villacoublay, France).

A linear elastic-brittle material was used in the FEM model. The following damage criterion was used: “max. principal stress”, damage evolution type—energy, softening–linear. According to [44], the size of the FEM mesh is of negligible importance in the X-FEM method. In order to confirm the validity of this thesis for the considered issue, an analysis of the sensitivity of the model to the size of the finite element mesh was carried out.

Simulation assumptions:

Type of material: 

**Sandston**: Elastic, Isotropic. Elastic Modulus—E =14,276 MPa, Poisson’s ratio—*ν* = 0.247, Tensile Strength—*σ*_t_ = 7.4 MPa, 

**Steel**–material: Elastic, Isotropic, Elastic Modulus—E = 210,000 MPa, Poisson’s Ratio—*ν* = 0.3.

*Damage initiation* in rock material: Maximal Principal Stress,

*Damage evolution*: type: Energy, Softening: Linear. Damage for traction separation Laws: Maximal Principal Stress Damage, Fracture Energy = 0.335 N/mm.

**Figure 6:**
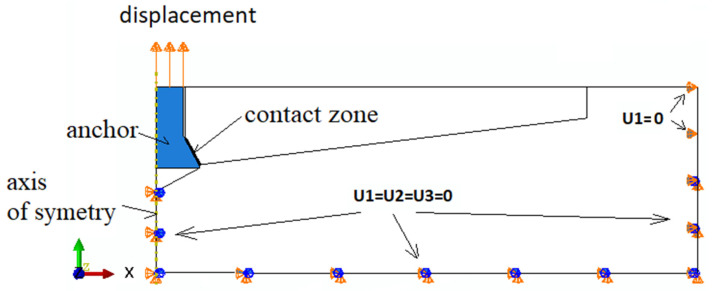
The scheme of boundary conditions assumed in the model.

The restraints/boundary conditions are shown in Figure 6. The nodes in the vertical axis of the model under the anchor and at the periphery of the model were deprived of all degrees of freedom (U1 = U2 = U3).

Interaction between rock and anchor: Interaction Type: Surface-To-Surface Contact (Standard), Discretization method: Surface to surface. Finite sliding. Applied minimal friction coefficient between rock and anchor material (steel)—*μ* = 0.2.

The anchor was subject to a controlled displacement (force) along the vertical axis (Figure 6). Between the conical head of the anchor and the rock, a contact was established along the cone’s forming line (ABAQUS–discretization method: surface to surface; finite sliding).

**Figure 7:**
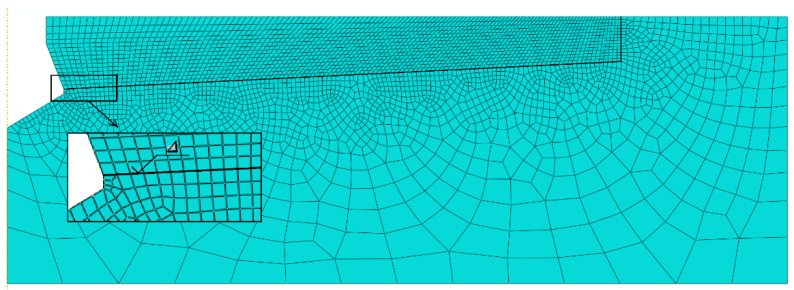
Discretization of a model with a finite element mesh; Δ—the length of the side of the mesh element measured at the side of the potential cone of destruction.

Finite elements were used (Figure 7):

*Sandston: Element type: CAX4R*: A 4-node bilinear axisymmetric quadrilateral, reduced integration, hourglass control. The finite element mesh was compacted in the region of the predicted potential rock fracture (Based on existing fracture penetration models existing in the subject literature).

*Anchor: Element type: CAX4R*.

The sensitivity of the model to the finite element mesh discretization method was evaluated using 6 mesh types [31]. The size of the mesh elements Δ was varied along the form of a hypothetical cone angle (inclined at an angle in the model, Figure 5 and Figure 7). Changes in the characteristic dimension Δ of the elements were made in 1 mm increments, ranging from 1 to 6 mm. The simulation results are illustrated in Figure 7.

**Figure 8:**
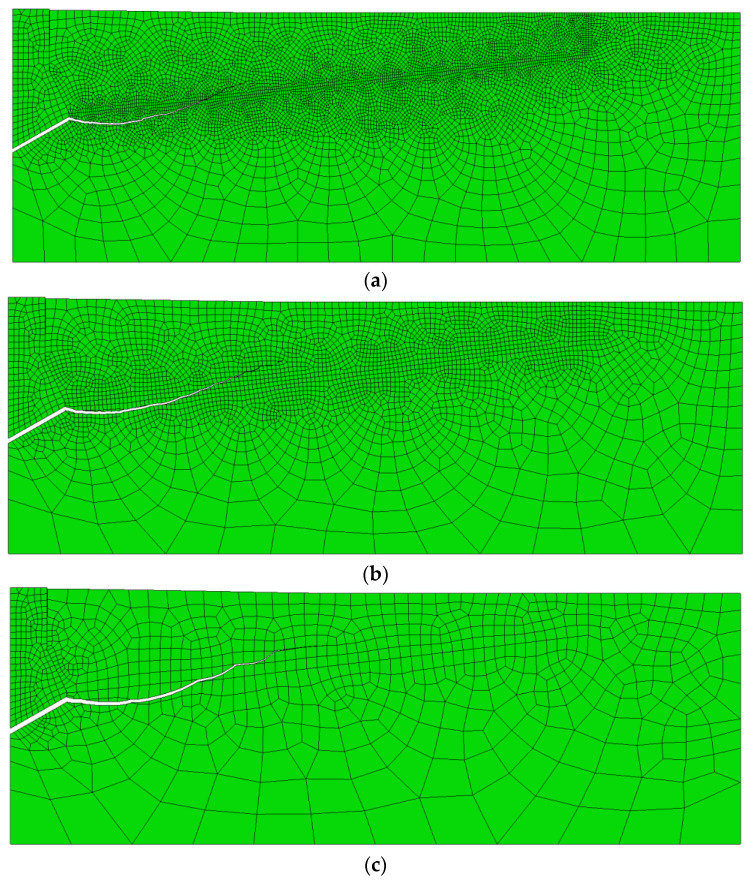
Model sensitivity to finite element mesh element size: (**a**) Δ = 1 mm, (**b**) Δ = 2 mm, and (**c**) Δ = 5 mm.

The analysis showed a strong correlation between the finite element mesh density in the model and the computation time, solution convergence and the quality of the failure trajectory representation. The model with component dimensions Δ = 1 mm requires a significantly longer computation time but makes it easier to obtain a “smooth” crack surface. The increase in the dimension Δ of the mesh elements causes a decrease in the accuracy of the gap representation. At the apex of the crack, the algorithm “stalls” and has a growing difficulty in correctly determining the potential crack direction of the material, leading to a halt in propagation. After evaluating the obtained solutions, it was decided to continue using the mesh with the characteristic dimension of the element on the side of the potential failure cone equal to Δ = 2 mm. Examples of model responses for the tested meshes are shown in Figure 8.

Finally, the parameters of anchor and rock in the rock–anchor contact were used for further analysis as follows:

Number of elements/nodes:

Rock: total number of nodes: 3325, total number of elements: 3240 linear quadrilateral elements of type CAX4R, reduced integration, hourglass control.

Anchor: element type CAX4R: a 4-node bilinear axisymmetric quadrilateral. Total number of nodes: 189, total number of elements: 160.

**Figure 9:**
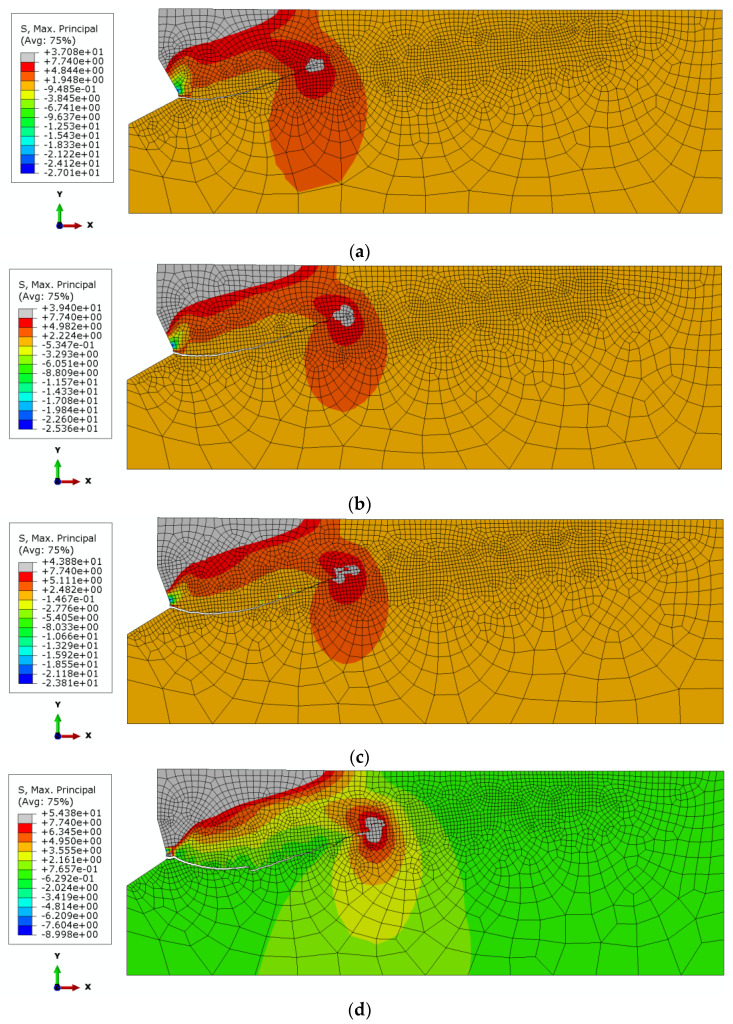
The initial fracture trajectory of the sandstone under the action of the undercutting anchor head, for *h*_ef_ = 50 mm, *μ* = 0.2 and head angle *β*: (**a**) *β* = 30°, (**b**) *β* = 25°, (**c**) *β* = 20°, and (**d**) *β* = 15°.

Figure 9 illustrates the distribution of the maximum principal stresses and the trajectories of the propagating fractures in the initial phase of the failure of the continuity of the rock medium for the considered cases of the angle of the worn out/extended head.

It is evident from Figure 9 that decreasing head angle *β*, being the effect of increasing wear of the anchor head, leads to an increase in crack penetration (in its initial stage of development) into the material, below the crack initiation point. This point, as can be seen from Figure 9, is located at the base of the undercutting head (lower edge of the cylindrical part with a height of 2 mm).

**Figure 10:**
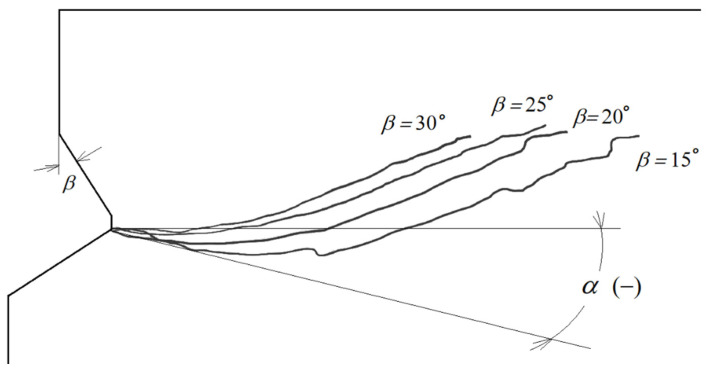
A comparison of sandstone fracture trajectories under anchor action at different stages of wear (summary) for equal anchor depths *h*_ef_ = 50 mm, (for *β* = 15° highest head wear–smallest undercutting head cone due to abrasive wear, *β* = 30°—the angle of the new head), and α—initial penetration angle of the failure surface.

The trend of changes in the shape and course of the crack/damage surface is clearly illustrated in Figure 10.

It is clear from Figure 10 that the initial joint penetration angle (α) for a new head (*β* = 30°) is close to 0°. Increasing the wear of the anchor head leads to a decrease in this angle, even to a value of −15°, for the most worn anchor head (*β* = 15°).

## 3. Experimental Verification

**Figure 11:**
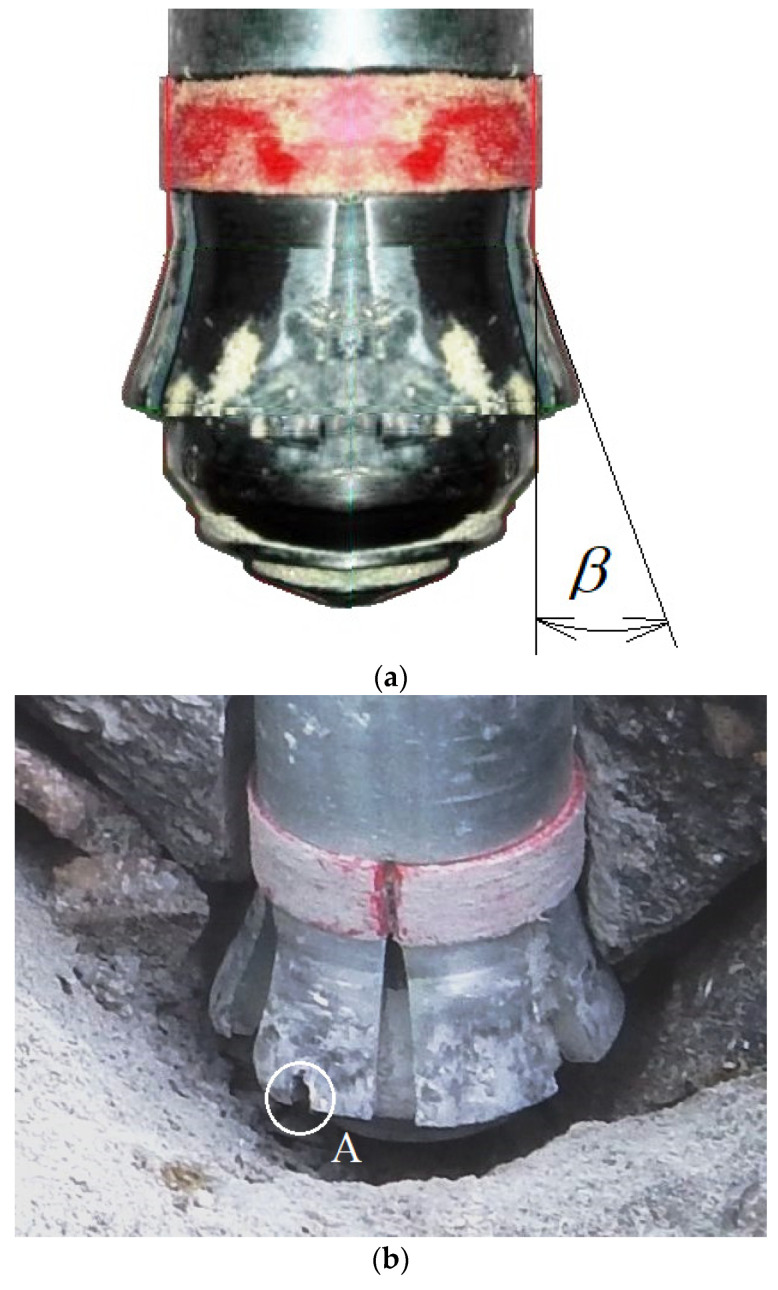
Symptoms of wear of the anchor head undercutting elements: (**a**) decrease in the angle of the head cone *β* and (**b**) reduction in the head angle due to removal of the tungsten carbide plate reinforcing the undercutting element—detail “A”.

**Figure 12:**
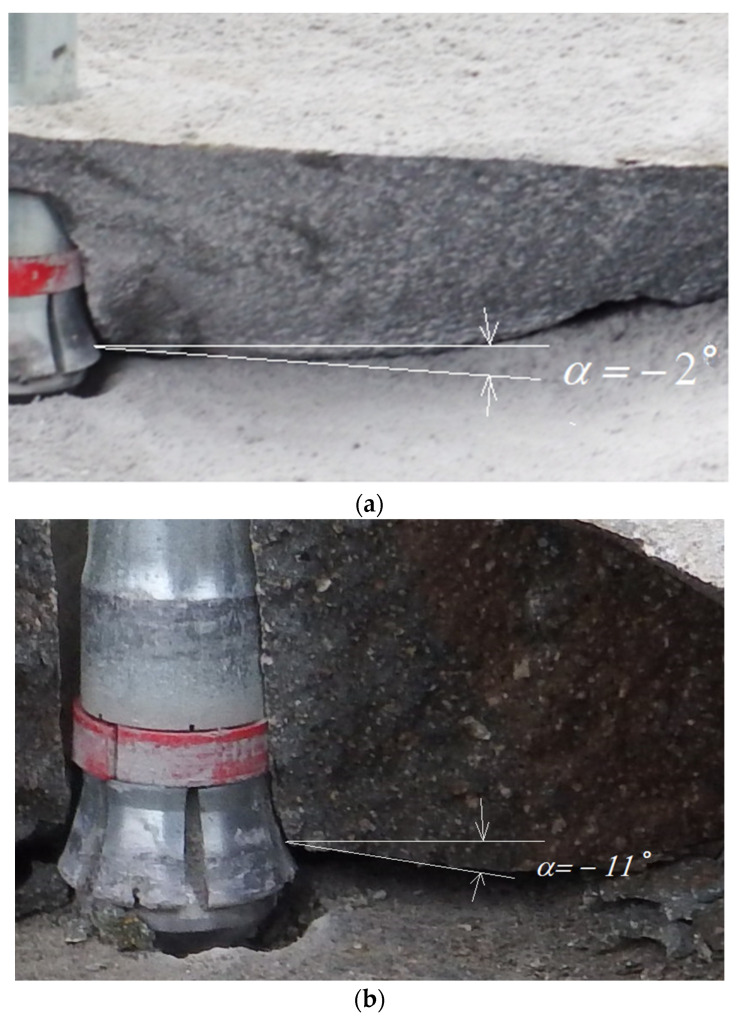
Initial propagation angle: (**a**) anchor head in early life *β* = 28° and (**b**) anchor head in advanced stage of wear, *β* = 19°, torn-off carbide reinforcement elements.

In the introductory part of the study, it was stated that symptoms of wear of the tested anchors were observed during field tests [26] (Figure 11). In extreme cases, carbide plates with which the head undercutting elements are equipped were also pulled out (Figure 10). As a result, the value of the cone angle *β* of the anchor head decreased significantly. This had an effect on the very different course and extent of the failure zone of the rock medium, especially on the initial angle (α, Figure 5) at which propagation of the crack/failure surface takes place. As a result, this leads to diversified shapes of detachments (Figure 12), decreasing initial angles of fracture propagation and increasing volumes of detached rock lumps [26].

As a result of the tests carried out within the project, 115 successful solid rock breakout trials were made using a fixed undercut anchor. Results for the Braciszów mine are summarized in Table 1. The Braciszów mine contained dense, compact sandstone.

The mechanical parameters of the rock used in the FEM simulation are within the range obtained in the strength tests summarized in Table 1.

Typical effects of the occurring wear of the conical surface of the undercutting anchor head are shown in Figure 11. The tendency to change the outline of the anchor head, intensive abrasion processes of the conical surface in the area of the base of the cone and the rounding of the edges of the undercutting elements are clearly visible. Figure 11b shows instances of critical forms of wear, i.e., the tearing out of the carbide inserts with which the cutting edges of the undercutting elements of the anchor head are currently reinforced (Hilti [9]). As a result, accelerated abrasion of the surfaces of the undercutting elements and a sudden change in the geometry of the entire head were recorded.

The field tests, therefore, confirmed the tendency observed in the FEM analysis for the angle of initial crack penetration (α) to decrease with progressive wear of the anchors. This process is illustrated in Figure 12.

## 4. Discussion

When analyzing the obtained results of numerical tests and field tests, one should take into account the simplifications assumed in the considerations. For example, the minimum value of the anchor head friction coefficient was assumed to be *μ* = 0.2. In actual mining conditions, the value of this coefficient may vary within wide limits, depending on the internal structure of the rock, its strength parameters or the degree of water saturation. Therefore, potential differences between the results of FEM simulations and those obtained in field tests may be attributed, among other things, to these phenomena.

Another aspect is the effective anchoring depth. According to the literature, for concrete, there is a significant dependence of the failure trajectory on this parameter. It is suggested that an increase in the anchoring depth translates into an increase in the failure cone angle α. This aspect was not considered in the numerical analysis.

A linear model of the rock medium was assumed in the numerical analysis. This was due to the fact that the algorithm for determining the direction of crack propagation, which is implemented in the ABAQUS program, in the case of the occurrence of I and II failure models, does not cope with solving this type of problem very correctly, e.g., [45]. As a result, the analysis of crack propagation can be carried out responsibly only in the range prior to the failure of the continuity of the rock medium. Therefore, it was assumed that the initial angle of crack propagation α will be analyzed. In order to fully illustrate the course of failure of a rock medium under the action of an undercut anchor, further analyses taking this aspect into account are necessary. 

Considering Figure 4, it can be seen that the head angle *β* has a significant effect on the distribution of forces on each of the undercutting elements of the undercutting anchor head. For the same value of rock-head friction angle (*ρ*, Figure 4) and the same value of pull-out force *F*’, decreasing head angle *β* causes an increase in the horizontal component *F*_x_. Between the rock friction factor *μ* considered in the model and the friction angle *ρ*, there is a relationship in the form of *tgρ*= *μ*. The FEM simulations show that for significant head wear (for small head angles) there are large deformations of the rock in the direction of the action of this component and a significant decrease in the value of the crack propagation initiation angle α (Figure 9 and Figure 10). This is also confirmed by the results of field tests (Figure 12). This may translate into an increase in circumferential stresses (in planes perpendicular to the head axis) favorable to faster rock failure through radial tearing). This issue was not analyzed due to the possibilities and limitations of the applied axisymmetric head model. 

Furche [41], Hirabayashi [46], and Thomson [42] showed that the value of the anchor head cone angle is very important for the failure process of the concrete structure as well as the formation of the anchor load capacity. However, they did not deal with the extent of the failure zone or the volume of the failure zone. However, it was found that the value of the head taper determines the specific stress state in the anchor–rock interaction zone, causing the production of specific components of the anchor pull-out force, leading to different concrete deformation (especially in the plane perpendicular to the anchor axis). For small head values, a large contribution of tensile circumferential stresses in the anchor–media contact zone has been demonstrated, leading to a faster destruction of the medium and a reduction in the anchor load capacity. The work of Jonak et al. [31] has shown a significant influence of the head angle on the formation of the detachment range (potentially the volume of detached solids). The value of the undercutting head angle depends to a large extent on the correctness of its installation and, in the case of the non-conventional technology of tearing off postulated by the authors, also on the wear process of the conical part of the undercutting head.

The results of numerical studies and field tests presented in this paper are a significant extension of the state of knowledge in terms of the impact of the shearing head on the rock, the course and range of the failure zone, and the influence of the head geometry on the process of rock medium destruction.

For mining applications of the proposed technology, attention should be paid to the wear rate of the anchor undercutting head. If the anchors are properly installed, decreasing the angle of the head as wear progresses can lead to an increase in the extent of the failure zone, a potential increase in the volume of rock lumps peeled off, and a decrease in the anchor pull-out force (for the same anchor depth). However, the influence of radial cracking on the considered parameters characterizing the anchor pull-out process requires further research using 3D FEM models and advanced material models of the rock medium. Further experiments and numerical studies are also required to determine the optimum configuration of anchor geometric parameters, anchor pull-out force (rock breaking energy) and the extent of the failure zone.

Despite the limitations of the numerical model used, the results of the analysis show quite clearly that increasing blunting of the undercutting anchor head (decreasing of angle *β* due to wear of the conical part of the head) leads to a clear change in the initial angle (α) of crack penetration (failure surface). This will potentially affect the extent of the failure surface and, therefore, the volume of the rock mass being peeled off, which is only approximate to a cone. A full explanation of this problem, however, requires the use of a more elaborate mechanical rock model, such as the Mohr–Coulomb or Drucker–Prager failure criterion, enabling analysis in terms of the covered development of fractures.

## Figures and Tables

**Table 1:** The mechanical characteristics of rocks in Braciszów mines.

*f*_c_ (MPa)	Standard Deviation	*f*_t_ (MPa)	Standard Deviation	k = f_c_/f_t_	*φ* (°)	*c* (MPa)	*E* (GPa)	Standard Deviation	ν (-)	Standard Deviation	*E*_f_ (N/mm)	Standard Deviation
155.3	29.17	7.614	0.64	19.41	49.5	14.5	15.745	4.757	0.203	0.068	0.329	0.049

*f*_c_—compressive strength; *f*_t_—tensile strength; *c*—cohesion; *φ*—angle of internal friction; k—strength asymmetry factor; *E*_f_—fracture energy; *ν*—Poisson’s Ratio.

## Data Availability

The data presented in this study are available on request from the corresponding author.
